# Rapidity of hematoma resolution after fibrinolytic therapy for intracerebral hemorrhage has a favorable effect on functional outcome

**DOI:** 10.1038/s41598-025-00469-6

**Published:** 2025-05-01

**Authors:** Vesna Malinova, Regina Schwiddessen, Christian von der Brelie, Dorothee Mielke, Veit Rohde

**Affiliations:** 1 Department of Neurosurgery, University Medical Center Göttingen, Göttingen, Germany; 2 Department of Neurosurgery, University Hospital Augsburg, Augsburg, Germany; 3 Department of Neurosurgery, Georg-August-University, Robert-Koch-Straße 40, 37075 Göttingen, Germany

**Keywords:** Intracerebral hematoma, Fibrinolytic therapy, Functional outcome, Neuroscience, Diseases of the nervous system, Stroke

## Abstract

Fibrinolytic therapy with tissue plasminogen activator (rtPA) is considered a promising treatment option for intracerebral hemorrhage (ICH), but a large randomized controlled study (i.e., MISTIE III) failed to show a benefit for the long-term outcome. This study investigated whether the rapidity of hematoma volume reduction influences outcome of ICH-patients undergoing fibrinolytic therapy. Patients with supratentorial ICH with or without a secondary extension to the ventricular system receiving fibrinolytic therapy from 2010 to 2020 were retrospectively analyzed. Patients with primarily intraventricular hemorrhage were excluded. A catheter was placed into the hematoma via burr hole and by means of neuronavigation. After confirming a correct catheter position rtPA was injected through the catheter with subsequent passive drainage of the hematoma. Hematoma volume was measured initially and 24/48/72 hours after treatment and the relative volume reduction was calculated. The functional outcome at discharge was assessed using the modified Rankin scale (mRS) regarding a mRS of 4 or lower as favorable outcome. A total of 280 patients with mean age of 69.6 years and mean hematoma volume of 55.6 ml were analyzed. The odds of reaching favorable outcome were four-fold higher in patients with a volume reduction of more than 50% after 24 h (OR 4.23, 95%CI 3.05 to 5.66, *p* = 0.007). Patients with a residual volume of less than 30 ml after 24 h had a two-fold higher chance of having favorable outcome (OR 2.9, 95%CI 1.78 to 4.63, *p* < 0.0001). A fast volume reduction of at least 50% within 24 h resulted into a favorable outcome in ICH-patients undergoing fibrinolytic therapy. Not just the amount but also the rapidity of hematoma volume reduction seems to be an important factor for a good clinical result after fibrinolytic therapy.

## Introduction

Intracerebral hemorrhage (ICH) is associated with a high mortality of up to 40% leaving the survivors of the bleeding with a substantial morbidity^1,2^. The initial brain damage is followed by a secondary brain injury induced by the mass effect of the hematoma on the surrounding brain parenchyma and the hematoma degradation products. The result of these processes is the development of perihematomal ischemia and edema^3^. Microsurgical hematoma evacuation via craniotomy was regarded as a promising treatment option for ICH intending to reduce the extent of brain damage and ameliorate morbidity. However, two randomized controlled trials failed to demonstrate outcome benefit of surgery^4–6^. Surgery-associated brain tissue damage outweighing the expected surgical benefit was deemed to be responsible for this finding. Subsequently, several minimally invasive surgical approaches for hematoma volume reduction have been developed and expected to be superior to hematoma evacuation via craniotomy^7,8^. In a recently published randomized controlled study (ENRICH trial), a minimally invasive surgical technique for early hematoma evacuation within 24 h after symptom onset was evaluated in comparison to best medical treatment alone. After enrolling 300 patients, 69% of them with lobar hematoma and 31% with a hematoma within the anterior basal ganglia, a functional benefit was shown at 180 days for minimally invasive surgery of lobar hematomas^9^. Fibrinolytic therapy with recombinant tissue-type plasminogen activator (rtPA) was evaluated in three randomized trials, that confirmed the feasibility and safety, but failed to show an outcome benefit^10–12^. The timing of intervention and the residual hematoma volume at the end of treatment have been used as explanations, why fibrinolytic therapy was not able to improve outcome despite of implementing less invasive surgical approaches. While several thresholds for residual volume at the end of treatment have been proposed, the rapidity of hematoma volume reduction remained a largely unexplored factor in this context^13^. The rapidity of ICH volume reduction may play a crucial role for the efficacy of fibrinolytic therapy, which was the rationale for performing this study. The hypothesis was that a faster ICH volume reduction within the first 24 h will result into a more favorable outcome at discharge setting the basis for further improvement during the following rehabilitation. This study investigated whether the rapidity of ICH volume reduction has an influence of functional outcome at discharge in ICH-patients undergoing fibrinolytic therapy.

## Methods

### Patient population

This is a retrospective observational study including consecutive patients with spontaneous supratentorial ICH, who fulfilled the criteria for receiving fibrinolytic therapy and were consequently treated with fibrinolytic therapy in the time from 2010 to 2020. Patients receiving hematoma evacuation via craniotomy, conservatively treated patients, patients with traumatic ICH or secondary ICH caused by a vascular pathology or tumor were excluded. Patients with primarily intraventricular hemorrhage without intraparenchymal hemorrhage were not included in the study. The ICH was diagnosed by performing non-enhanced computed tomography (CT) within 24 h after symptom onset. Whenever secondary ICH was suspected due to clinical or imaging characteristics, a computed tomography angiography (CTA) or magnetic resonance imaging (MRI) was additionally carried out to confirm or exclude secondary pathologies. All procedures were in accordance with the ethical standards of the responsible committee on human experimentation and with the Helsinki Declaration. An approval was obtained for this study from the Ethics Committee of the University Medical Center Göttingen (Number 3/11/20). Due to the retrospective study design, an informed consent was waived by the Ethics Committee of the University Medical Center Göttingen. No study specific interventions were performed.

### Surgical procedure for performing fibrinolytic therapy

Fibrinolytic therapy was indicated in patients with primary ICH without underlying secondary pathology with an ICH volume of at least 30 ml and clinical impairment resulting into a Glasgow coma scale (GCS) of less than 14 points. The ICH score was used to make the decision to perform fibrinolytic therapy or not^14^. Patients with ICH score 2–5 are considered candidates for fibrinolytic therapy, where patients with ICH score 6 were not considered candidates for fibrinolytic therapy due to a high 30-day mortality rate of 100% despite treatment^15^. The decision for conducting fibrinolytic therapy was made by the neurosurgeon on duty applying the selection criteria as stated above. All patients included in the study were either directly admitted to our hospital after symptom onset or were transferred to our hospital from near smaller clinics directly after performing a CT scan confirming an ICH. A CT scan was repeated after four hours to confirm clot stability followed by the intervention. The estimated time duration between ICH onset and surgery was under twelve hours. In case of an ongoing anticoagulation, a reversal of anticoagulation was initiated by administrating prothrombin complex concentrate with a threshold for the Quick test value of > 60%. No platelet transfusion was conducted in patients with antithrombotics in the patients included in this study. A small skin incision and a burr hole were performed followed by the placement of catheter into the hematoma along the longest diameter using the navigation system (Brainlab^®^, Feldkirchen, Germany). A CT scan was repeated directly after the procedure to confirm a correct catheter position within the hematoma before starting the fibrinolytic therapy. In two patients the position of the catheter was revised directly after the CT scan, where the following CT scan confirmed a correct intrahematomal position. The rtPA (Actilyse^®^ Boehringer Ingelheim Pharma GmbH & Co. KG, Ingelheim am Rhein, Germany) dosage was calculated depending on the size of the hematoma with administering 1 mg per 1 cm hematoma diameter as previously described^16^. The rtPA was injected on one to three consecutive days starting with the day of ICH diagnosis, which was congruent with the day of symptom onset. The time duration between surgery and the first rtPA injection was on average one hour. After rtPA injection the drainage was clamped for 30 min and then reopened with drainage of the liquefied hematoma against negative pressure gradient. Approximately 24 h after each rtPA administration a CT scan was performed. Whenever a relevant residual hematoma (> 15 ml) was present, and the catheter position was still within the hematoma a further rtPA dosage was injected up to two times.

### Volumetric analysis

The hematoma volume was initially measured as well as 24/48/72 hours after treatment using the object creation application of the Brainlab^®^ software (Elements, Brainlab^®^, Feldkirchen, Germany). The relative volume reduction was calculated after 24, 48, and 72 h after treatment, respectively. A hematoma volume reduction of > 50% within the first 24 h or a hematoma volume of < 30 ml (since hematomas with a volume of > 30 ml are regarded as hematomas requiring surgical intervention) were considered as a cutoff to relieve the space occupying effect of the hematoma. Additionally, a hematoma volume reduction of < 15 ml was evaluated according to the cutoff defined in the MISTIE III trial.

### Outcome measures

The primary outcome was the functional outcome, which was evaluated using the modified Rankin scale (mRS) at discharge (6 = death). The mRS at discharge was performed by the physician at the intensive care unit, who was responsible for the discharge of the patient. The assessment was done in person. A mRS ≤ 4 at discharge was considered sufficiently good outcome with the potential of further clinical improvement after rehabilitation. The rationale for setting the cutoff at mRS 4 was to choose a reasonably achievable outcome at discharge in this patient collective that can improve during rehabilitation to a more desirable outcome of mRS 3 or smaller in the long-term. Secondary outcome parameters were rebleeding and a surgical site infection.

### Statistical analysis

The statistical analyses were performed by means of the GraphPad Prism software (Version 9, GraphPad Software, San Diego, CA, USA). For the presentation of baseline data descriptive statistics and frequency distribution analysis was done. Continuous variables are depicted as mean ± standard deviation (SD), categorical variables as frequency or percentages. Not normally distributed variables were reported using median and interquartile range (IQR). Regression analysis was done for identification of factors associated with a faster hematoma resolution and a good outcome. Fisher’s exact test was performed to calculate odds ratios (OR). Multivariate regression analysis was performed to identify independent outcome predictors. The dependent variable was the outcome at discharge according to mRS. Age, GCS on admission, ICH volume on admission, IVH, ICH location, and hematoma volume reduction of > 50% after 24 h were significant outcome predictors in the univariate analysis and, hence, were included in the multivariate analysis to identify the independent outcome predictors.

## Results

### Patient characteristics

A total of 280 ICH-patients treated with fibrinolytic therapy were analyzed. The mean age was 69.6 ± 12.5 years, and 46% (129/280) of patients were male. In 54% (150/280) of patients the hematoma was located within the basal ganglia, and 46% (130/280) of patients had a lobar hematoma. Arterial hypertension was present in 66% of all patients. More than the half of patients (55%) had an anticoagulation (Marcoumar^®^ = phenprocoumon) and/or an antiaggregating medication (AspirinⓇ = acetylsalicylic acid, and/or PlavixⓇ = clopidogrel) at manifestation. All included patients in the study population had an intraparenchymal hemorrhage, that was extended to the ventricular system with presence of intraventricular hemorrhage in 70.7% (198/280) of patients. In 63 of patients with additional presence of intraventricular hemorrhage an external ventricular drainage was placed to treat acute hydrocephalus. The median ICH score in the study population was 3 (IQR 2–4), and the mean initial ICH volume was 55.6 ± 25.6 ml. The baseline characteristics of the study population are summarized in Table 1.

**Table 1:** Baseline characteristics of the study population.

Parameters	
Number of patients	280
Mean age (SD) in years	69.6 (12.5)
Sex	
Male	46% (129/280)
Female	54% (151/280)
Mean initial ICH-volume (SD) in ml	55.6 (25.6)
ICH location	
Lobar hematoma	46% (130/280)
Deep-seated hematoma	54% (150/280)
ICH-score	
ICH-score 1	8% (22/280)
ICH-score 2	25% (70/280)
ICH-score 3	39% (110/280)
ICH-score 4	24% (67/280)
ICH-score 5	4% (11/280)
Arterial hypertension	
Yes	66% (182/280)
No	34% (93/280)
Blood thinner	
Heparin	4% (11/280)
Phenprocoumon	22% (60/280)
Direct oral anticoagulants	5% (15/280)
Thrombocytes antiaggregation	24% (66/280)
No blood thinner	45% (128/280)

### Rapidity of ICH volume reduction with fibrinolytic therapy

**Fig. 1:**
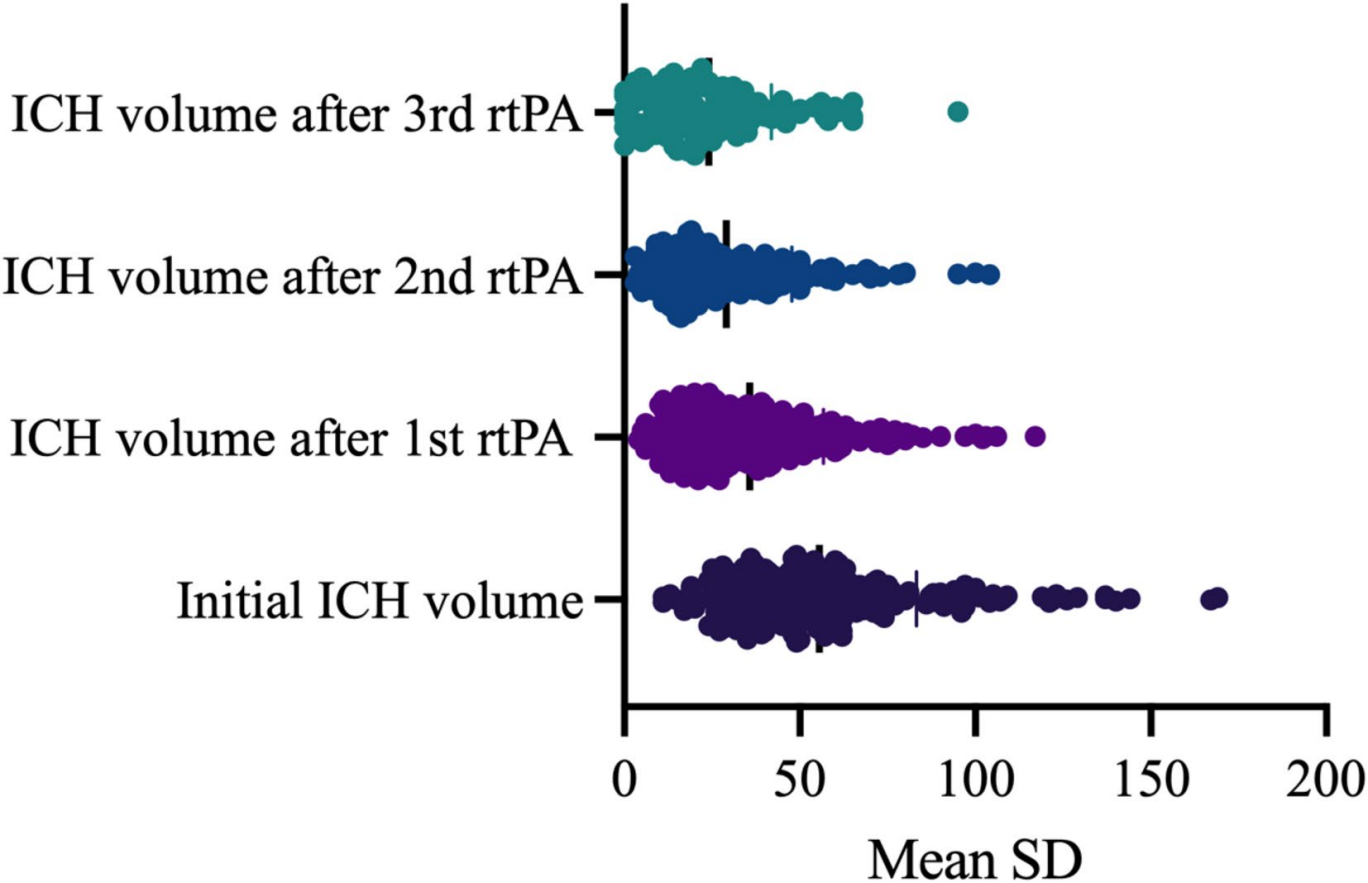
Average hematoma volume reduction over time during fibrinolytic therapy.

Three rtPA dosages were administered in 39% (109/280), two rtPA dosages in 36% (101/280) of all patients, whereas 25% (70/280) of the study population received only one rtPA dosage. The first rtPA injection has the highest contribution to the overall relative volume reduction (median 50%, 95%CI 37–58%), while the contributions of the second rtPA dose (median 17%, 95%CI 13–20%), and the third rtPA injection (median 12%, 95%CI10-15%) were significantly smaller (*p* < 0.0001), Fig. 1. The median residual hematoma volume was 32 ml (95%CI 28–35 ml) after 24 h, 24 ml (95%CI 21–28 ml) after 48 h, and 20 ml (95%CI 18–25 ml) after 72 h. The median relative volume reduction in the study population was 34% (95%CI 31–37%) after 24 h, 51% (95%CI 47–55%) after 48 h, and 64% (95%CI 57–67%) after 72 h. A hematoma volume reduction of at least 50% after 24 h (volume reduction after the first rtPA dosage) was found in 28% (77/280) of the study population. There was no significant difference of the initial hematoma volume between the group with ≥ 50% within 24 h and the group with < 50% hematoma clearance (mean 55.7 ml vs. 55.2 ml, t-test, *p* = 0.89). In 5% (14/280) of patients a relative volume reduction higher than 70% was achieved after 24 h. A residual hematoma volume < 30 ml was found in 46% (130/280) 24 h after treatment. A residual hematoma volume of ≤ 15 ml had 27% (96/280) of patients 48 h after treatment and 36% (101/280) 72 h after treatment. There was a negative correlation of ≥ 50% relative hematoma volume reduction with the presence of any kind of blood thinner in the medication of the patents on admission (*r*= – 0.13, 95%CI – 0.24 to – 0.01, *p* = 0.03), and no correlation with the initial hematoma volume (*r* = – 0.007, 95%CI – 0.12 to 0.10, *p* = 0.89), the hematoma location (*r* = – 0.003, 95%CI – 0.15 to 0.08, *p* = 0.54), the hematoma’s shape (*r* = 0.09, 95%CI – 0.02 to 0.20, *p* = 0.13), and the catheter position within the hematoma (*r* = 0.02, 95%CI – 0.09 to 0.14, *p* = 0.71).

### ICH volume reduction and impact on functional outcome at discharge

**Fig. 2:**
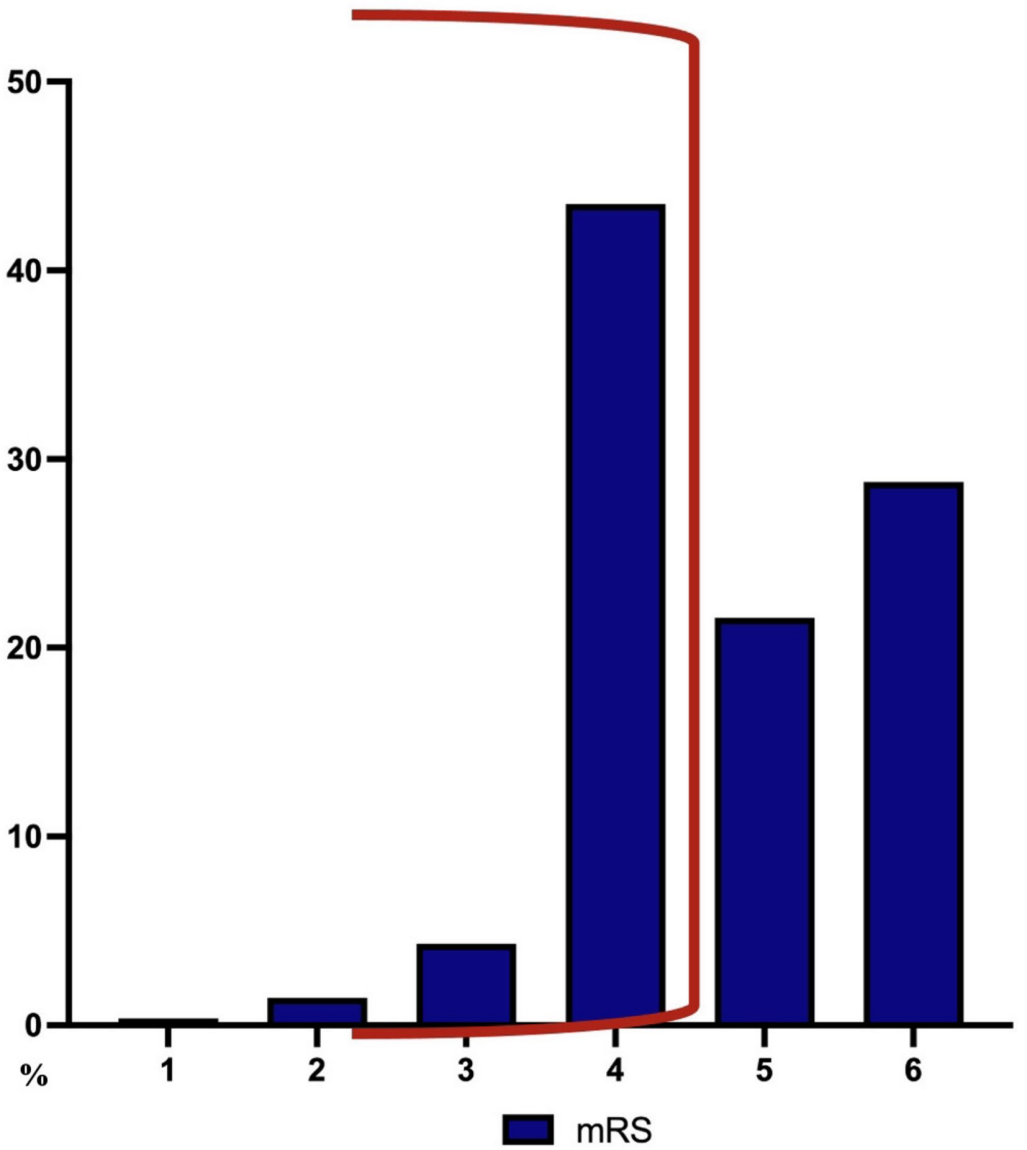
Functional outcome according to modified Rankin scale at discharge after fibrinolytic therapy.

**Fig. 3:**
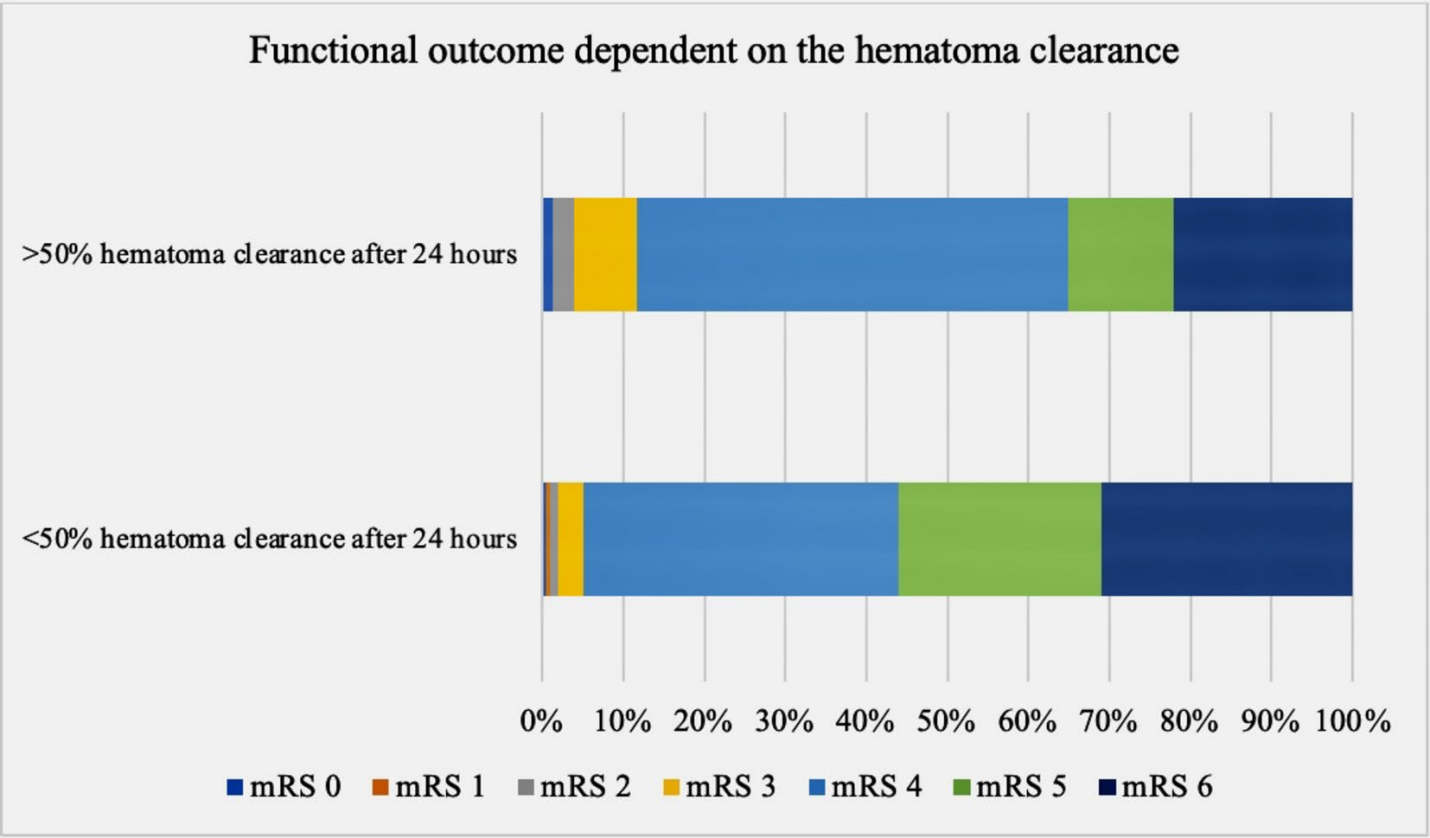
Improvement in functional outcome at discharge in patients with faster hematoma resolution with > 50% relative hematoma volume reduction within 48 h after ictus (lower diagram) compared to their counterparts with slower hematoma resolution (upper diagram).

A mRS ≤ 3 had only 7% (19/280) of all patients, 58% (11/19) of whom were transferred to a rehabilitation facility, 37% (7/19) were discharged at home, and 5% (1/19) were transferred to a short-term care facility. A mRS of four was found in 43% (121/280) of all patients, 90% (108/121) of whom were discharged to a rehabilitation facility, 7% (9/121) were transferred to a near to home hospital, and 3% (4/121) were transferred to a nursing home (Fig. 2). A mRS of five was found in 21% (60/280) of all patients, 75% of whom were transferred to a rehabilitation facility, 13% were admitted in a palliative department, and 12% were transferred to a nursing home. The in-hospital mortality (mRS 6) in the study population was 29% (80/280). The odds of reaching a mRS of four at discharge was four-fold higher in patients with a relative volume reduction of at least 50% after 24 h compared to patients with a relative volume reduction of less than 50% (OR 4.23, 95%CI 3.05 to 5.66, *p* = 0.007). There was no positive impact on functional outcome at discharge (OR 1.68, 95%CI 0.63 to 2.01, *p* = 0.22), when a relative volume reduction of at least 50% was achieved after 48 h or later. A mRS ≤ 3 at discharge was also more often achieved in the patient group with a relative volume reduction of at least 50% after 24 h compared to the patient group with a relative volume reduction of < 50% after 24 h, but the difference did not reach statistical significance (*p* = 0.06), Fig. 3. Patients with < 30 ml residual hematoma after 24 h had a two-fold higher chance of reaching mRS ≤ 4 at discharge (OR 2.9, 95%CI 1.78 to 4.63, *p* < 0.0001). Patients with < 15 ml residual hematoma after 48 h had a three-fold higher chance of reaching mRS ≤ 4 at discharge (OR 3.6, 95%CI 2.55 to 5.01, *p* < 0.0001). There were no cases of rebleeding or a surgical site infection in the study population. In 9 out of the 63 patients with additional placement of external ventricular drainage a cerebrospinal fluid infection was documented and treated with antibiotics. The multivariate regression analysis revealed age, GCS on admission, initial hematoma volume, presence of IVH, and a hematoma volume reduction of > 50% after 24 h to be independent outcome predictors at discharge (Table 2).

**Table 2:** Multivariate analysis of outcome predictors at discharge with the outcome at discharge assessed according to mRS as the dependent variable.

Variables	Estimate	Standard error	95% CI	ItI	*P* value
Age	0.0106	0.0042	0.0023 to 0.0190	2.526	0.01
GCS on admission	− 0.1377	0.0153	− 0.1679 to − 0.1075	8.976	< 0.0001
ICH volume on admission	0.0061	0.0019	0.0022 to 0.0100	3.127	0.002
IVH	0.2874	0.1153	0.0603 to 0.5145	2.492	0.01
ICH location deep vs. lobar	0.1367	0.1097	− 0.0793 to 0.3527	1.245	0.21
> 50% hematoma volume reduction after 24 h	− 0.2479	0.1141	− 0.4725 to − 0.02337	2.174	0.03

GCS = Glasgow Coma Scale, ICH = intracerebral hemorrhage, IVH = intraventricular hemorrhage.

## Discussion

Currently available treatments of ICH are directed to reduction of secondary brain injury and amelioration of morbidity. Thus, an early and fast hematoma evacuation is deemed to have the potential of diminishing and possibly even preventing ongoing pathophysiologic processes leading to secondary brain injury after ICH. Fibrinolytic therapy facilitates a hematoma resolution and a consecutive hematoma drainage with a low risk profile for surgery-associated complications and held promise to improve outcome. Nevertheless, fibrinolytic therapy failed to meet this expectation in several previously published randomized controlled trials^10,12^. A benefit with a reduction of the mortality rate was seen whenever a residual volume of ≤ 30 ml and an overall volume reduction of > 53% was achieved^17^. The treatment goal of the MISTIE trial was to achieve an ICH volume reduction to 15 ml residual hematoma at the end of treatment (one day after the last rtPA dose administration) or until a maximum of 9 doses of rtPA (1 mg every 8 h) were administered. This treatment goal has been achieved in 59% of included patients in the MISTIE trial^10,17^. The timing of surgical intervention for ICH has been already evaluated in the MISTIE trial and the STICH I and II trials suggesting that earlier surgery does not necessarily result into better long-term outcome^5,6,13^. The rapidity of hematoma volume reduction, however, has not been directly addressed as an efficacy determinator of fibrinolytic therapy in previously published studies. In this study, we addressed this issue and were able to demonstrate a clear impact of ICH volume reduction rapidity on reached outcome at discharge. Patients with faster ICH volume reduction of at least 50% of initial hematoma volume within 24 h had significantly higher odds of achieving better outcome.

### Parameters affecting the efficacy of fibrinolytic therapy

A meticulous patient selection is crucial for an effective fibrinolytic therapy. According to the MISTIE criteria, patients with lobar or deep-seated supratentorial hematomas with an initial ICH volume of more than 30 ml, Glasgow Coma Scale score of ≤ 14 points, symptom onset within 24 h of diagnosis, were considered eligible for fibrinolytic therapy^10^. In the past years, further factors such as hematoma shape and appearance on initial CT scan were reported to influence the efficacy of fibrinolytic therapy, that were not considered in the MISTIE trials. Irregularly shaped hematomas were associated with larger hematoma, intraventricular hemorrhage, with less efficient ICH removal, severe disability at discharge, and death^18,19^.

### Timing of fibrinolytic therapy and functional outcome

Modified Rankin scale is the most frequently used outcome assessment tool in stroke patients, where mRS of 0–3 is usually regarded as favorable outcome, which has been reported in 33–37% of patients with ICH at 12-month follow-up^20,21^. Functional independence (mRS 0–2) at 6-months follow-up has been reached in 27% of patients with ICH undergoing endoscopic hematoma evacuation^22^. In our study population, only 7% of patients had mRS 0–3 at discharge, questioning the cutoff mRS 0–3 as target for outcome assessment in ICH patients at discharge. A mRS cutoff of 4 seems to be a more representable target to be reached by ICH patients at discharge with the potential for further recovery during rehabilitation. Since long-term follow-up data after rehabilitation were not available, we cannot prove this hypothesis based on our data. However, previous studies reported a recovery potential of ICH patients with mild and moderately severe disability at the beginning of rehabilitation, which supports our hypothesis^23^. Another issue, that needs to be mentioned in this context, is the use of different assessment tools for functional independency in different studies preventing a direct comparison^21^. Furthermore, considering the disability paradox, that has been found among stroke patients, there are several other factors affecting the quality of life in these patients, that are not considered by most outcome assessment tools leading to a limited prediction of a desirable outcome in this patient group when relying on these scores alone^24,25^. A balanced consideration of these factors is necessary for setting outcome measure targets in ICH trials and defining the outcome status worth surviving the bleeding. Factors associated with higher odds of reaching independency were a better clinical status at manifestation, a lack of intraventricular hemorrhage, and a shorter time (< 48 h) from symptom onset to evacuation^22,26^. In a recently published study, time to endoscopic hematoma evacuation was independently associated with good long-term functional outcome (mRS 0–3 at 6-months follow-up), where for every additional hour there was a 5% reduction in the odds of achieving a favorable outcome^27^. Furthermore, the findings of the ENRICH trial evaluating the impact of minimally invasive surgical evacuation of intracerebral hematoma within 24 h after onset revealed a functional benefit after 180 days for lobar hematomas compared to best medical treatment alone^9^. The impact of timing of intervention on functional outcome was evaluated in the study populations of the STICH trials and the MISTIE III trial, where 71% of included patients underwent the MISTIE procedure between 24 and 72 h, and 82% of patients enrolled in the STICH trials underwent surgery ≤ 48 h. The authors found similar therapeutic window of 47 h from ictus to surgery beyond which worse outcome were documented^28^. The mean residual hematoma volume was 28.8 ml in the MISTIE trial and 30 ml in the STICH trial. Although, several studies have reported the importance of residual volume or volume reduction threshold and timing from ictus to surgical intervention, the effect of hematoma volume reduction celerity remained unexplored. After performing a comprehensive volumetric analysis following each rtPA injection in this study, a significantly better outcome could be demonstrated in patients with substantial (> 50%) volume reduction within 24 h compared to their counterparts with a slower volume resolution.

### Limitations of the study

The retrospective study design is the main limitation of the study. The decisions to perform fibrinolytic therapy were made on the discretion of the attending neurosurgeon. Missing data concerning the long-term functional outcome is a limitation of the study. Furthermore, this is a monocentric study based on a study population treated at our hospital, that impedes a generalization of the findings. However, the study involves a large consecutive patient cohort treated with fibrinolytic therapy including a comprehensive volumetric analysis, which is a strength of the study.

## Conclusion

A fast volume reduction of at least 50% of the initial ICH volume within 24 h resulted in a favorable outcome in ICH patients undergoing fibrinolytic therapy. Not just the amount of hematoma reduction but also the rapidity of volume reduction seems to be an important criterium for an effective fibrinolytic therapy. These findings support the hypothesis that the rapidity of hematoma volume reduction has a significant influence on the functional outcome in ICH-patients undergoing fibrinolytic therapy by limiting the toxicity of blood degradation products.

## Data Availability

All generated and analyzed data is already presented in the manuscript.
